# Supplementary material for: The preclinical inhibitor GS441524 in combination with GC376 efficaciously inhibited the proliferation of SARS-CoV-2 in the mouse respiratory tract
Source: Emerg Microbes Infect. 2021 Mar 19;10(1):481–92. doi: 10.1080/22221751.2021.1899770 (PMC7993387; doi:10.1080/22221751.2021.1899770)
Supplement: Supplementary_information.docx [file TEMI_A_1899770_SM0390.docx]

**Supplementary Information for:**

**The Preclinical Inhibitor GS441524 in Combination with GC376 Efficaciously Inhibited the Proliferation of SARS-CoV-2 in the Mouse Respiratory Tract**

Yuejun Shi^1,3#^, Lei Shuai^2#^, Zhiyuan Wen^2^, Chong Wang^2^, Yuanyuan, Yan^1,3^, Zhe Jiao^1,3^, Fenglin Guo^1,3^, Zhen F. Fu^1,3^, Huanchun, Chen^1,3^, Zhigao Bu^2,4*^, Guiqing Peng^1,3, *^

1 State Key Laboratory of Agricultural Microbiology, College of Veterinary Medicine, Huazhong Agricultural University, Wuhan, China

2 State Key Laboratory of Veterinary Biotechnology, Harbin Veterinary Research Institute, Chinese Academy of Agricultural Sciences, Harbin 150069, China

3 Key Laboratory of Preventive Veterinary Medicine in Hubei Province, The Cooperative Innovation Center for Sustainable Pig Production, Huazhong Agricultural University, Wuhan, China

4 National High Containment Laboratory for Animal Diseases Control and Prevention, Harbin 150069, China

# These authors contributed equally to this work

*Correspondence to Zhigao Bu (buzhigao@caas.cn) and Guiqing Peng ([penggq@mail.hzau.edu.cn](mailto:penggq@mail.hzau.edu.cn))

1. **Supplemental Tables**

**Supplemental Table1 Data collection and refinement statistics**

| Parameter | Value^a^ | |
| --- | --- | --- |
|  | 7CBT |  |
| Data collection statistics |  |  |
| Space group | P 1 21 1 |  |
| Cell parameter(a, b, c (Å)) | 55.12, 99.75, 60.36. |  |
| α, β, γ (°) | 90.00°, 108.63°, 90.00° |  |
| Wavelength (Å) | 1.5418 |  |
| Resolution (Å) (range) | 45.00-2.35 |  |
| No. of reflections | 1103121 |  |
| Completeness (%) | 96.1 (99.9) |  |
| R_merge_^b^ (%) | 15.0 (56.10) |  |
| I/Sigma (last shell) | 17.6 (2.6) |  |
| Redundancy (last shell) | 5.0 (4.5) |  |
|  |  |  |
| Refinement statistics |  |  |
| Resolution (Å) (range) | 28.15-2.35 |  |
| No. of reflections | 25716 |  |
| R_work_/R_free_^c^(%) | 21.0/29.2 |  |
| No of protein atoms | 4616 |  |
| No. of solvent atoms | 177 |  |
| RMSD |  |  |
| Bond length (Å) | 0.016 |  |
| Bond angle (°) | 1.57 |  |
| Average B factor (Å^2^) | 50.0 |  |
| Ramachandran plot: core,  allow, disallow | 93.24%, 4.90%, 1.86% |  |

^a^ The highest-resolution values are indicated in parentheses.

^b^ R_merge_=∑∑∣I_i_-﹤I﹥∣/∑∑I_i_; where is I_i_ the intensity measurement of reflection h and﹤I﹥is the average intensity from multiple observations.

^c^ R_work_=∑||F_o_|-|F_c_|| /∑|F_o_|; where F_o_ and F_c_ are the observed and calculated structure factors, respectively; R_free_ is equivalent to R_work_, but 5% of the measured reflections have been excluded from the refinement and set aside for cross-validation.

1. **Supplemental Figures**


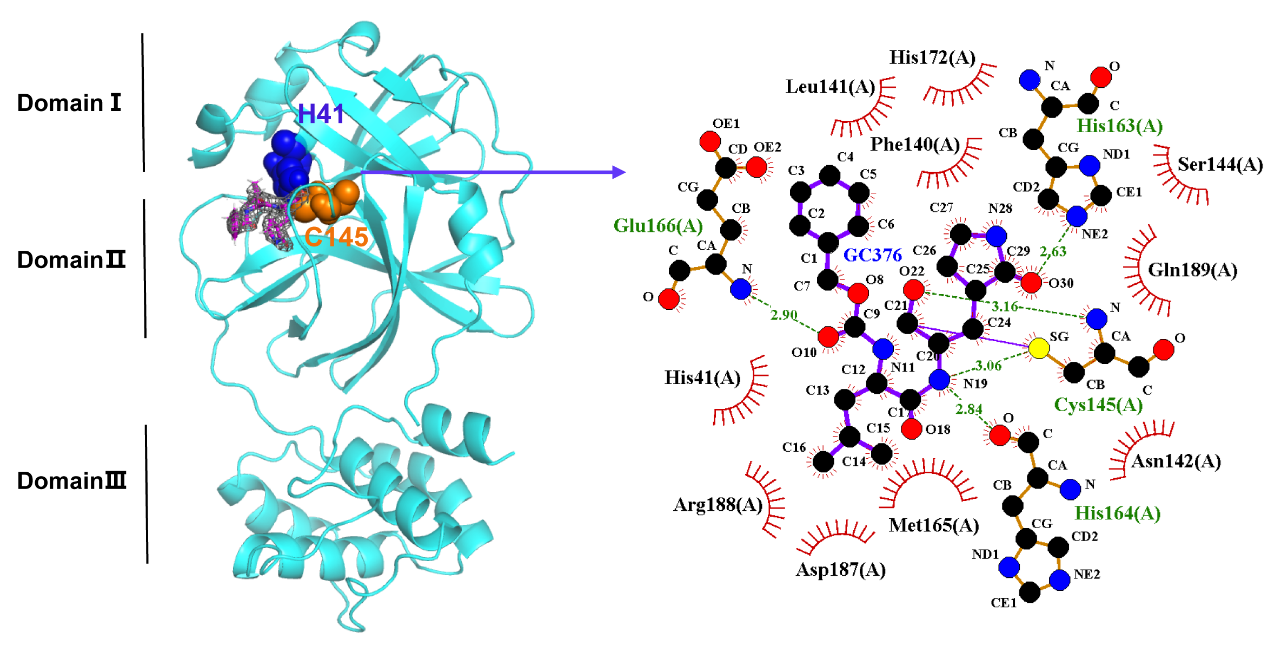


**Supplementary Figure 1 Interaction analysis of GC-376 targeting SARS-CoV-2 M^pro^.** The interacting residues between GC376 and M^pro^ were determined using LIGPLOT. Carbon, nitrogen, oxygen and sulfur atoms are shown as black, blue, red and yellow circles, respectively. Hydrogen bonds are shown as green dashed lines labeled with the distance between the donor atom and corresponding acceptor atom. Hydrophobic interactions are demonstrated by arcs with spokes radiating toward the atoms (with spokes around) or residues (shown as arcs with spokes) they contact.


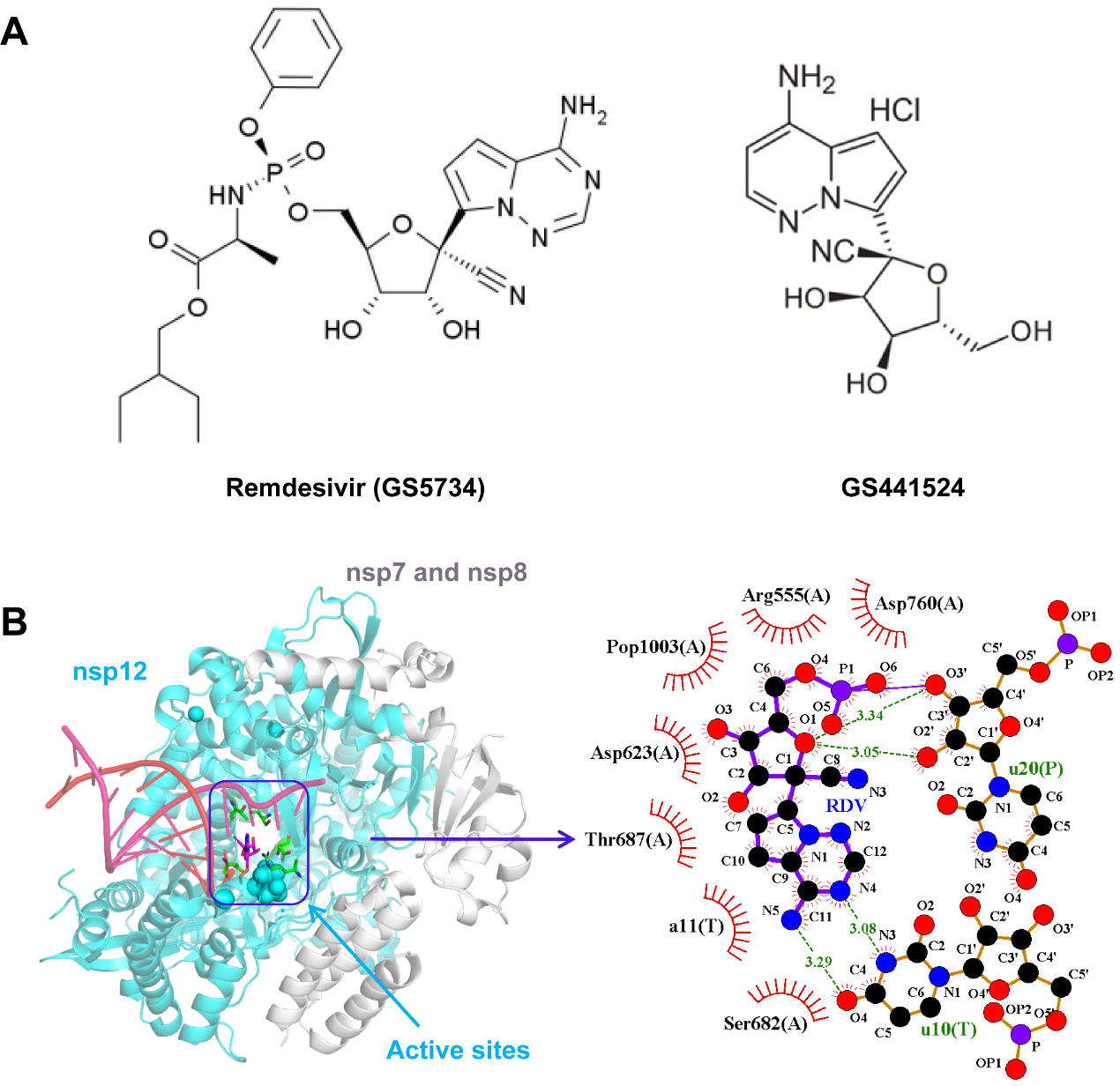


**Supplementary Figure 2 Interaction analysis** **of GS441524 targeting SARS-CoV-2 RdRp.** (A) The chemical structures of remdesivir (RDV, GS5734) and GS-441524. (B) The interacting residues between RDV and RdRp were determined using LIGPLOT. Carbon, nitrogen (or phosphorus) and oxygen atoms are shown as black, blue and red circles, respectively. Hydrogen bonds are shown as green dashed lines labeled with the distance between the donor atom and corresponding acceptor atom. Hydrophobic interactions are demonstrated by arcs with spokes radiating toward the atoms (with spokes around) or residues (shown as arcs with spokes) they contact.


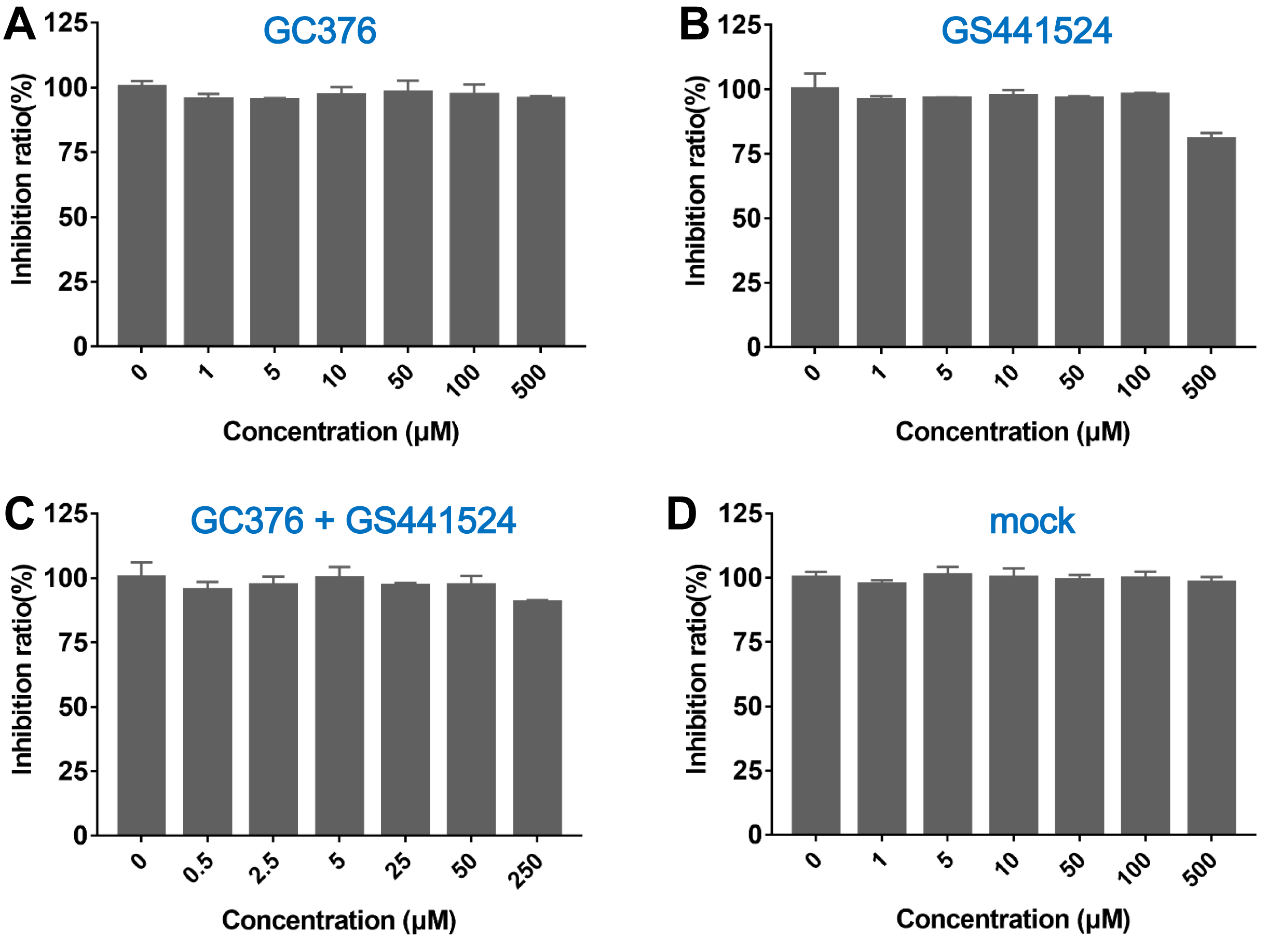


**Supplementary Figure 3 Cell viability analysis of GC376, GS441524 and GC376+GS441524 in Vero E6 cells.** Cell viability was determined 24 h post-inoculation (GC376 and GS441524, 0-500 μM; GC376+GS441524, 0-250 μM) by using the Cell Titer-Glo kit following the manufacturer’s instructions. The error bars show the S.D. of the results from three replicates.

**
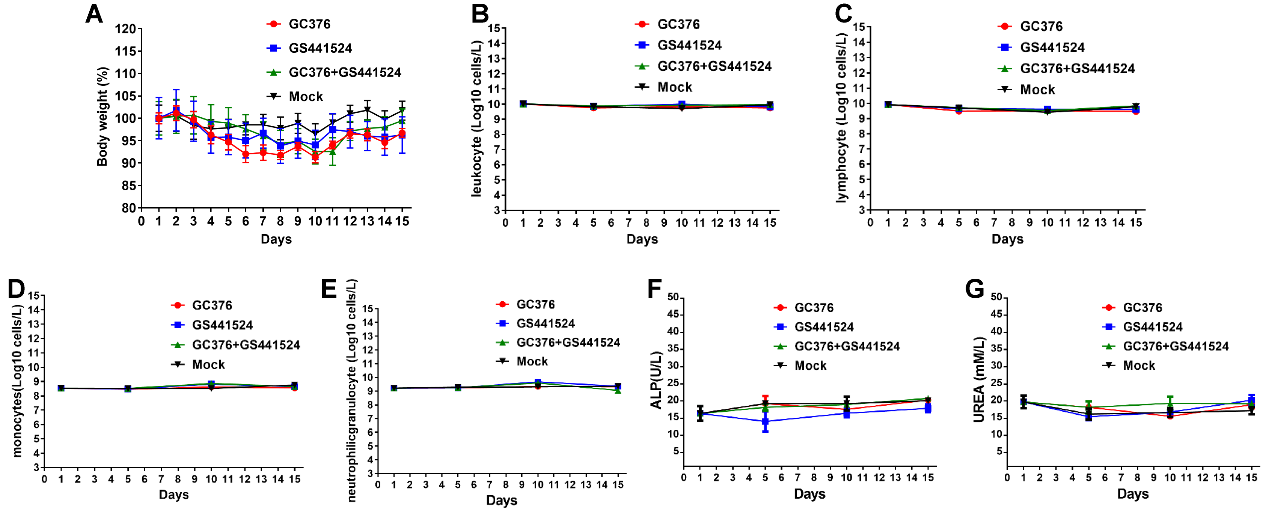
**

**Supplementary Figure 4 Body weight, blood cell counts and blood chemistry measurements in mice intramuscularly administered GC376 and GS441524 in the safety study.** (A) Body weights of mice over time. Dose range toxicity studies were performed for 15 days. (B-G) Various blood cell counts or blood chemistry values over time are shown. ALP, alkaline phosphatase. UREA, carbamide. The data were analyzed using GraphPad Prism 7.0, and the error bars show the SEM of the results from five replicates.


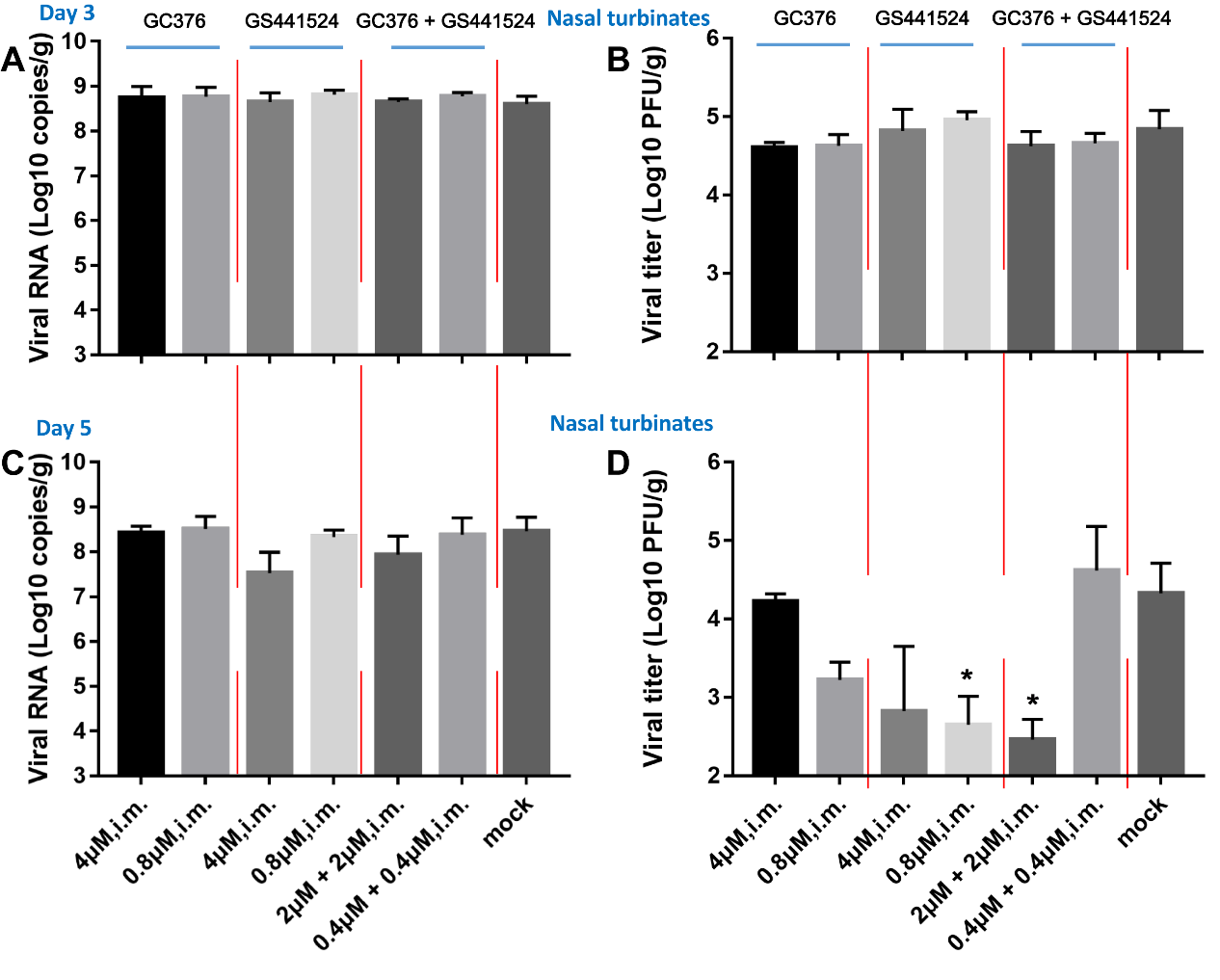


**Supplementary Figure 5** **Evaluation of i.m. GC376, GS441524 and GC376+GS441524 against SARS-CoV-2 infection in mouse nasal turbinate.** Four- to six-week-old female BALB/c mice were intramuscularly administered a loading dose of GC376 (4 or 0.8 µM), GS441524 (4 or 0.8 µM), GC376 + GS441524 (2 µM+2 µM or 0.4 µM+0.4 µM), followed by a corresponding daily maintenance dose. Control mice were administered vehicle solution (12% sulfobutylether-β- cyclodextrin, pH 3.5) daily, in parallel (0 µM). One hour after administration of the loading dose of GC376, GS441524, GC376 + GS441524 or vehicle solution, the mice were inoculated intranasally with 10^3.6^ PFU of HRB26M in a volume of 50 μl. On days 3 and 5 p.i., three mice in each group were euthanized and their nasal turbinates and lungs were collected. Viral RNA copies and infectious titers in the nasal turbinates (A-D) were detected using qPCR and viral titration. The concentrations of the daily maintenance doses are shown. The data were analyzed using GraphPad Prism 7.0, and the error bars show the SEM of the results from three replicates. *P < 0.05 (considered significant) compared to the viral RNA copies or infectious titers of the mock-treated group.


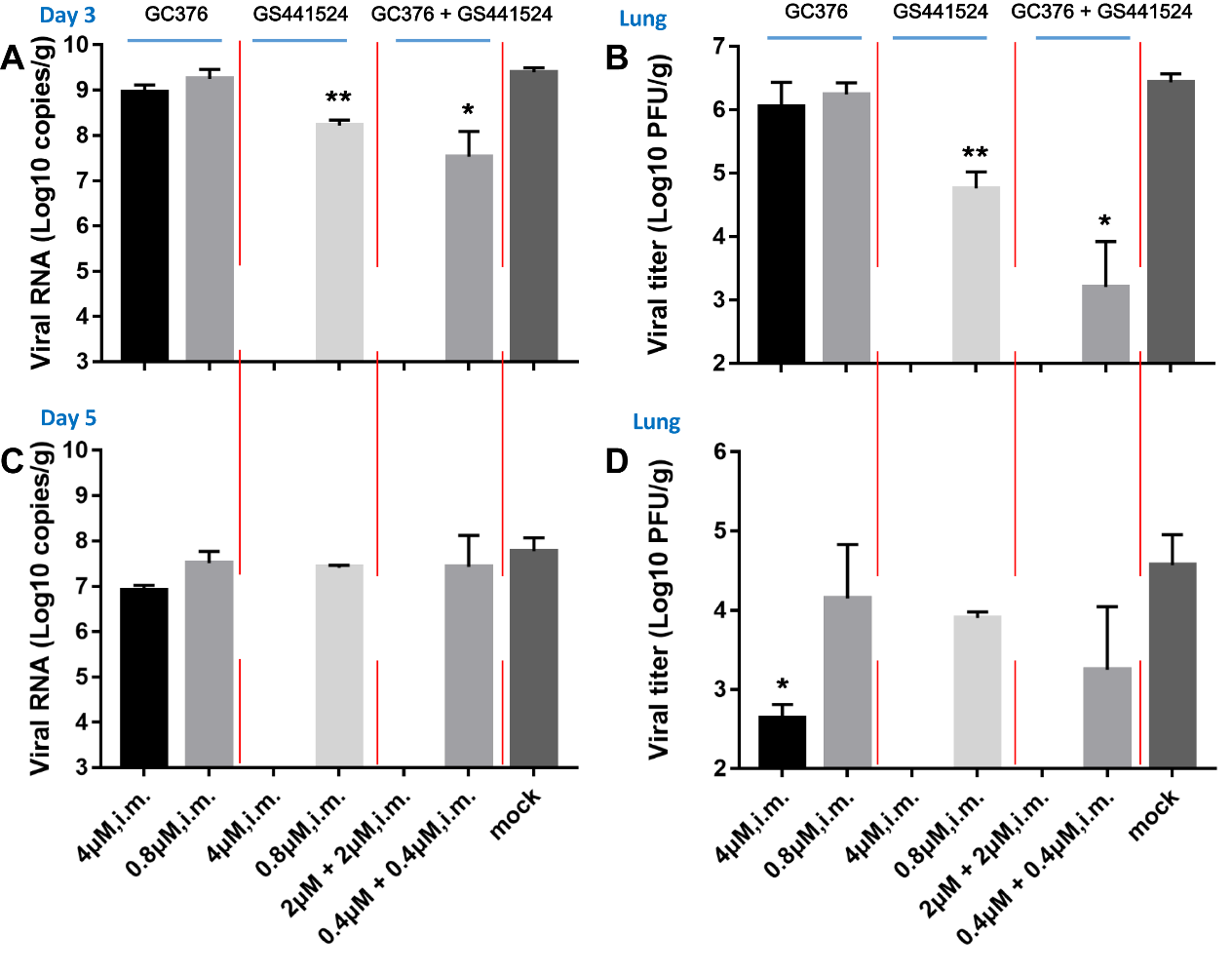


**Supplementary Figure 6 Evaluation of i.m. GC376, GS441524 and GC376+GS441524 against SARS-CoV-2 infection in mouse lung.** Detailed description is the same as Supplementary Figure 5. Viral RNA copies and infectious titers in the lungs (A-D) were detected using qPCR and viral titration. The concentrations of the daily maintenance doses are shown. The data were analyzed using GraphPad Prism 7.0, and the error bars show the SEM of the results from three replicates. *P < 0.05 (considered significant) compared to the viral RNA copies or infectious titers of the mock-treated group; **, P < 0.01 (considered highly significant).


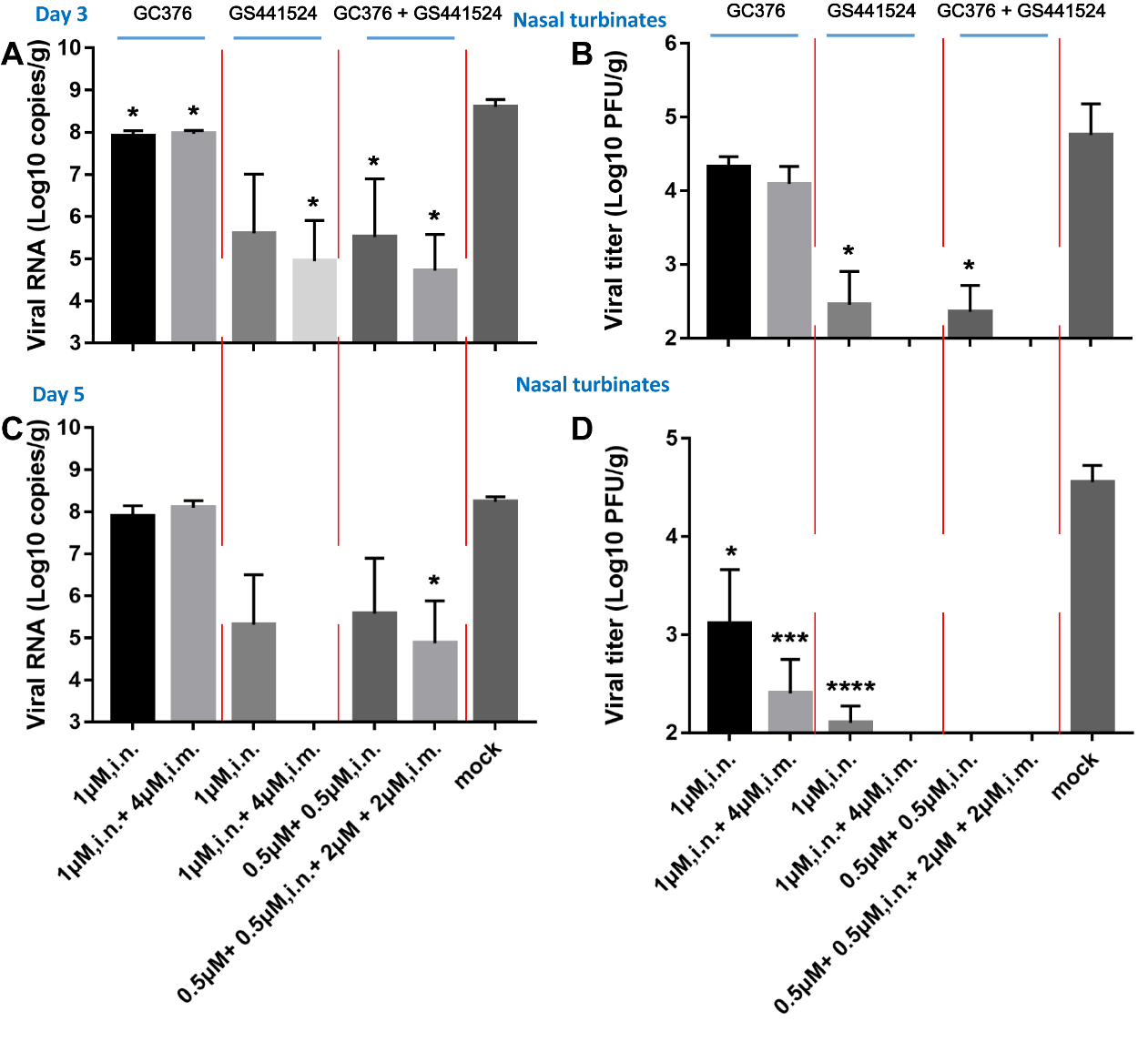


**Supplementary Figure 7 Evaluation of i.n. and i.m. GC376, GS441524 and GC376+GS441524 against SARS-CoV-2 infection in mouse nasal turbinate.** Four- to six-week-old female BALB/c mice were administered a loading dose of GC376 (1 µM, i.n. or 1 µM, i.n.+4 µM, i.m.), GS441524 (1 µM, i.n. or 1 µM, i.n.+4 µM, i.m.), GC376 + GS441524 (0.5 µM+0.5 µM, i.n. or 0.5 µM+0.5 µM, i.n. and 2 µM+2 µM, i.m.), followed by a corresponding daily maintenance dose. Control mice were administered vehicle solution (12% sulfobutylether-β- cyclodextrin, pH 3.5) daily in parallel (0 µM). One hour after administration of the loading dose of GC376, GS441524, GC376 + GS441524 or vehicle solution, the mice were inoculated intranasally with 10^3.6^ PFU of HRB26M in a volume of 50 μl. On days 3 and 5 p.i., three mice in each group were euthanized and their nasal turbinates and lungs were collected. Viral RNA copies and infectious titers in the nasal turbinates (A-D) were detected using qPCR and viral titration. The concentrations of the daily maintenance doses are shown. The data were analyzed using GraphPad Prism 7.0, and the error bars show the SEM of the results from three replicates. *P < 0.05 (considered significant) compared to the viral RNA copies or infectious titers of the mock-treated group; **, P < 0.01 (considered highly significant); ***, P < 0.001 or ****, P < 0.0001 (considered extremely significant).


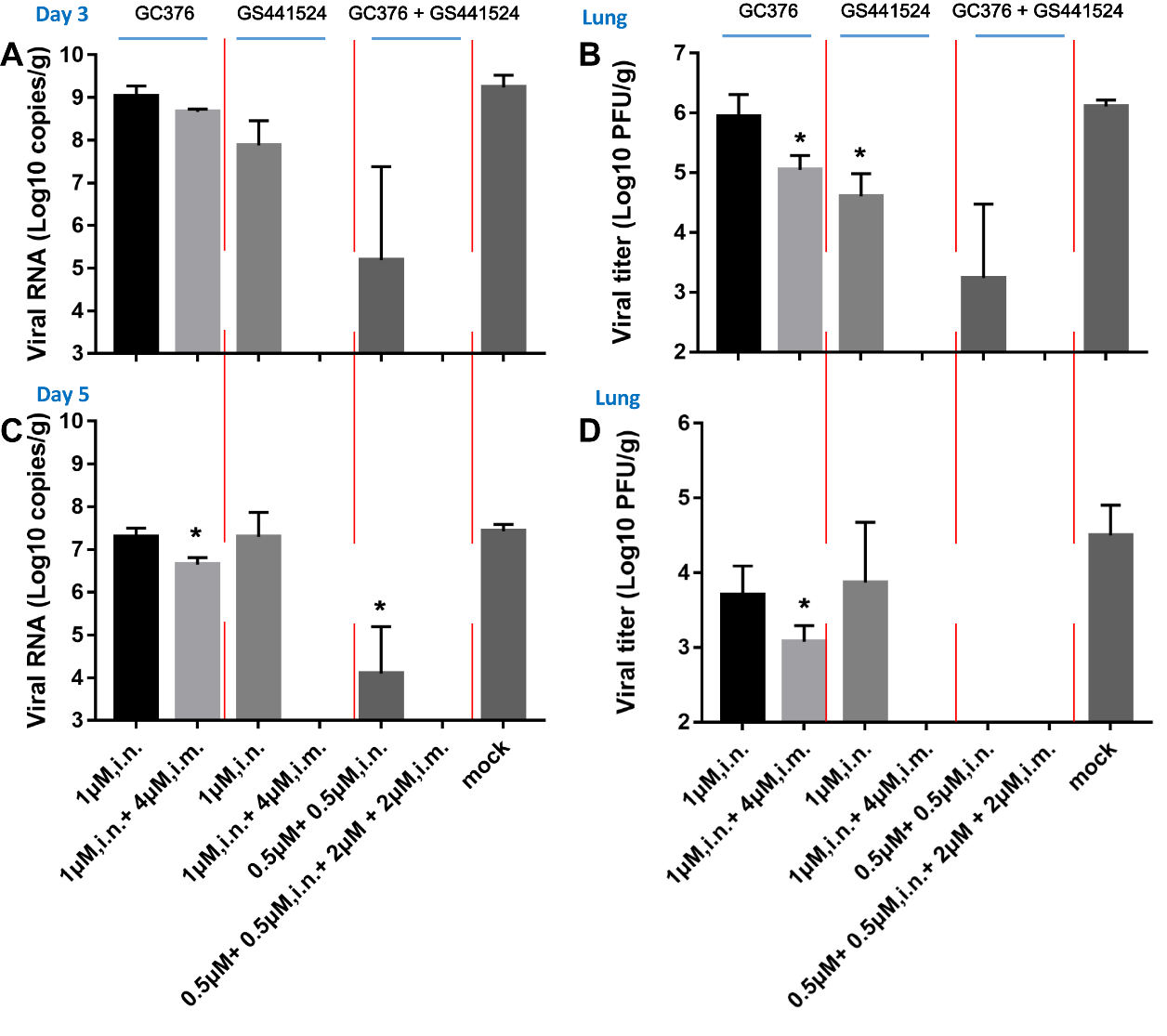


**Supplementary Figure 8 Evaluation of i.n. and i.m. GC376, GS441524 and GC376+GS441524 against SARS-CoV-2 infection in mouse lung.** Detailed description is the same as Supplementary Figure 7. Viral RNA copies and infectious titers in the lungs (A-D) were detected using qPCR and viral titration. The concentrations of the daily maintenance doses are shown. The data were analyzed using GraphPad Prism 7.0, and the error bars show the SEM of the results from three replicates. *P < 0.05 (considered significant) compared to the viral RNA copies or infectious titers of the mock-treated group.
